# Supplementary material for: The Systems Biology Research Tool: evolvable open-source software
Source: BMC Syst Biol. 2008 Jun 29;2:55. doi: 10.1186/1752-0509-2-55 (PMC2446383; doi:10.1186/1752-0509-2-55)
Supplement: Additional file 1 — SBRT Archive. An archive of the current version of the Systems Biology Research Tool. [file 1752-0509-2-55-S1.zip › sbrt-1.4.0/doc/developers_guide/api/sbrt/kernel/math/graph_theory/proc/CycleIdProcess.html]

CycleIdProcess


|  |  |  |
| --- | --- | --- |
| **Overview** \| **Package** \| **Class** \| **Use** \| **Tree** \| **Deprecated** \| **Index** \| **Help** | |  |
| PREV CLASS   **NEXT CLASS** | **FRAMES**    **NO FRAMES**     **All Classes** |  |
| SUMMARY: NESTED \| FIELD \| CONSTR \| METHOD | DETAIL: FIELD \| CONSTR \| METHOD |  |


---


## sbrt.kernel.math.graph\_theory.proc Class CycleIdProcess<N>

```
java.lang.Object
  sbrt.kernel.math.graph_theory.proc.CycleIdProcess<N>
```

**Type Parameters:**: `N` - the node type.

**All Implemented Interfaces:**: KernelProcess<Path<N>>, MonitoredProcess<Path<N>>

---

``` public class CycleIdProcess<N> extends java.lang.Object implements MonitoredProcess<Path<N>> ```

This class is used to identify all the simple cycles in a directed graph.

**Author:**
:   This class was written and documented by Jeremiah Wright
    while in the Wagner lab.

---

**Constructor Summary**

`CycleIdProcess()`
  
          Constructs a new cycle-identification experiment.


| **Method Summary** | |
| --- | --- |
| `ProgressMonitor` | `getProgress()`             Returns a progress monitor for this experiment. |
| `void` | `run(ResultsManager<Path<N>> resultsManager)`             Adds to the provided results manager each identified cycle in the current graph. |
| `void` | `setGraph(DirectedGraph<N> graph)`             Sets the graph for which all cycles will be identified. |

**Methods inherited from class java.lang.Object**

`clone, equals, finalize, getClass, hashCode, notify, notifyAll, toString, wait, wait, wait`

**Constructor Detail**

### CycleIdProcess

```
public CycleIdProcess()
```

:   Constructs a new cycle-identification experiment.


**Method Detail**

### setGraph

```
public void setGraph(DirectedGraph<N> graph)
```

:   Sets the graph for which all cycles will be identified.

    :   **Parameters:**: `graph` - the graph for which all paths will be identified.

---


### getProgress

```
public ProgressMonitor getProgress()
```

:   Returns a progress monitor for this experiment.

    :   **Specified by:**: `getProgress` in interface `MonitoredProcess<Path<N>>`
    :   **Returns:**: a progress monitor for this experiment.

---


### run

```
public void run(ResultsManager<Path<N>> resultsManager)
```

:   Adds to the provided results manager each identified cycle in the current
    graph.

    :   **Specified by:**: `run` in interface `KernelProcess<Path<N>>`
    :   **Parameters:**: `resultsManager` - the results manager to which the cycles are added.


---


|  |  |  |
| --- | --- | --- |
| **Overview** \| **Package** \| **Class** \| **Use** \| **Tree** \| **Deprecated** \| **Index** \| **Help** | |  |
| PREV CLASS   **NEXT CLASS** | **FRAMES**    **NO FRAMES**     **All Classes** |  |
| SUMMARY: NESTED \| FIELD \| CONSTR \| METHOD | DETAIL: FIELD \| CONSTR \| METHOD |  |


---
